# The Convergence of Precision and Cognition in Biomedical AI

**DOI:** 10.3390/bioengineering13030264

**Published:** 2026-02-25

**Authors:** Luis Pinto-Coelho, João Paulo Teixeira, João Paulo Carmo

**Affiliations:** 1 ISEP, Polytechnic of Porto, 4249-015 Porto, Portugal; 2 INESC TEC, 4200-465 Porto, Portugal; 3 Research Centre in Digitalization and Intelligent Robotics (CEDRI), Instituto Politécnico de Bragança, Campus de Santa Apolónia, 5300-253 Bragança, Portugal; joaopt@ipb.pt; 4 Department of Electrical Engineering (SEL), University of São Paulo (USP), Avenida Trabalhador São-Carlense, nr. 400, São Carlos 13566-590, SP, Brazil; jcarmo@sc.usp.br

## 1. Introduction

The historical trajectory of biomedical engineering has been defined by the pursuit of enhanced resolution, whether in the spatial granularity of an MRI scan, the temporal precision of a kinematic sensor, or the spectral clarity of an audio recording. However, as we navigate the third decade of the 21st century, the defining challenge has transitioned from mere data acquisition to that of data cognition. The exponential growth of biomedical data has outpaced the human capacity for manual interpretation, necessitating a paradigm shift wherein algorithms do not simply process signals but interpret, reason, and interact with them. This Special Issue, “Machine Learning-Driven Innovations in Biomedical Signal and Image Processing”, captures a crucial moment in this evolutionary process [1,2], presenting a collection of research that spans the full spectrum of modern artificial intelligence (AI), from the methodical feature engineering of evolutionary algorithms to the emergent reasoning capabilities of Agentic AI.

The five articles selected for this Editorial synthesis represent a small sample of the current state of the art. They address a range of diverse clinical domains, including rectal cancer staging, Parkinson’s disease monitoring, respiratory diagnostics, and radiological reporting. Nevertheless, they are unified by a common methodological aspect: the move towards automated, robust, and clinically interpretable machine learning systems. We observe a dual evolution in this field: On one hand, there is a refinement of “classical” deep learning and radiomics, where the focus is on standardisation and biological plausibility. On the other, we see the introduction of Large Language Models (LLMs) and Vision–Language Models (VLMs), which threaten to disrupt traditional diagnostic workflows by integrating semantic understanding into the domain of signal processing.

This Editorial explores these innovations through three thematic lenses: the evolution of imaging biomarkers and automated curation; the advancement of signal processing in kinematics and acoustics; and the emerging frontier of Agentic AI in mitigating generative errors. These works demonstrate that the future of biomedical signal processing lies in the intelligent synthesis of multimodal data, bringing together pixel, sensor, waveform, and language data.

## 2. The Visual Frontier: From Radiomics to Vision–Language Alignment

In the domain of medical imaging, the extraction of quantitative features, known as radiomics, has long promised to unveil biological characteristics imperceptible to the human eye. However, the translation of these models into clinical practice still reveals methodological heterogeneity.

The systematic review and meta-analysis by Gómez et al. [3] addresses a critical clinical need: the preoperative prediction of pathological nodal status in rectal cancer. Accurate staging is the cornerstone of treatment planning, distinguishing early-stage disease from locally advanced cases requiring neoadjuvant therapy. While high-resolution MRI is the gold standard for T-staging, identifying metastatic lymph nodes remains a significant challenge due to the overlap in appearance between reactive and malignant nodes.

The authors analysed 16 studies encompassing over 3000 patients, evaluating the efficacy of MRI-based radiomics. The findings revealed a landscape of promise tempered by variability. The pooled sensitivity of 0.68 and specificity of 0.73 indicate that while radiomics models demonstrate moderate accuracy, they are not yet infallible replacements for histopathology. A striking finding is the potential of diffusion-weighted imaging (DWI); while T2-weighted images were universally used, models incorporating DWI often yielded superior performance, with one study achieving an Area Under the Curve (AUC) of 0.92. This suggests that functional imaging features, which capture tissue cellularity, may offer more discriminative power than morphological features alone.

However, this review highlights a persistent barrier to clinical translation: heterogeneity. The variations in the MRI field strength (1.5 T vs. 3.0 T), the segmentation targets (tumour vs. lymph nodes vs. mesorectum), and the machine learning classifiers create a fragmented evidence base. Furthermore, the authors identify indications of publication bias, suggesting that smaller studies with negative results are under-reported, potentially inflating the perceived efficacy of radiomics. The editorial position here is clear: for radiomics to mature, the field must move beyond isolated, small-sample studies towards multi-centric validation with harmonised acquisition protocols.

While Gómez et al. [3] focus on the analysis of annotated data, Yamamoto and Kikuchi [4] address an antecedent bottleneck: the scarcity of annotated datasets themselves. The construction of large-scale medical visual question answering (MedVQA) datasets is hindered by the lack of explicit links between free-text radiology reports and specific 2D image slices in volumetric scans.

To bridge this gap, the authors propose a Contrastive Language–Image Pre-training (CLIP)-based key slice selector. Taking advantage of the joint embedding space of CLIP, they align report sentences with the most semantically relevant CT slices. This approach represents a shift from supervised learning on manually labelled data to self-supervised alignment of multimodal data. The study demonstrates that while general-domain CLIP models perform poorly, domain-specific variants like BiomedCLIP, pre-trained on the biomedical literature, achieve significantly higher accuracy.

The authors introduce a fine-tuning protocol that employs a “dual-supervised” dataset, thereby imparting both lesion awareness (the identification of slices exhibiting pathology) and organ awareness (the identification of normal anatomy). This fine-tuning procedure enhances the top 1 accuracy for lesion localization by over 20 percentage points in comparison to the baseline CLIP model. The clinical relevance of this method is substantiated through a radiologist review, which yields an acceptance rate of 56.32% for the automated slice selection. This innovation is transformative, as it facilitates the automated generation of massive, clinically realistic datasets from hospital archives, thereby effectively circumventing the bottleneck of manual annotation.

## 3. The Kinematic and Auditory Frontier: Biological Inspiration and Evolutionary Computing

Moving beyond static imaging, this Special Issue highlights significant advances in the processing of temporal signals, specifically, the kinematic data of Parkinson’s disease (PD) and the acoustic signatures of pulmonary health. In both domains, the papers demonstrate that successful classification depends heavily on how raw signals are transformed into interpretable features. This is an important step towards language-independent classifiers that can universally detect PD patterns [5].

Psathas et al. [6] present a novel framework for the objective assessment of PD motor symptoms using a custom-developed SmartGlove system. Parkinson’s disease is characterised by affected motor control, including tremor, bradykinesia, and rigidity, which are often difficult to quantify during brief clinical visits. The proposed glove is equipped with a nine-axis Inertial Measurement Unit (IMU) that can capture high-fidelity kinematic data during standard motor tasks.

The methodological core of this work is Grammatical Evolution (GE). Rather than relying solely on standard statistical features, the authors employ GE to synthetically construct “artificial features”. This process evolves mathematical expressions (using a Backus–Naur Form grammar) that combine raw inputs into non-linear representations optimised for classification. This is distinct from traditional deep learning approaches that treat feature extraction as a “black box”.

The results obtained show the benefits of this evolutionary approach. The generated features, often composites of non-linear dynamic measures like the Lyapunov Exponent and Higuchi Fractal Dimension, outperformed standard PCA and Neural Network classifiers, reducing classification errors to between 10% and 19%. The study found that Resting Tremor Observation was the most discriminative task, achieving error rates as low as 10.35% with the constructed features. This highlights the importance of non-linear dynamics in biological signal processing; the chaotic nature of pathological tremor is better captured by fractal dimensions and entropy measures than by simple variance or mean frequency. By employing a multi-method feature selection scheme that integrates statistical significance, model-based importance, and variance contribution, the authors ensured that the selected features were not only predictive but physiologically meaningful.

The proposed approach, while primarily validated for pharmacological monitoring, can also establish an essential framework for closed-loop therapeutic management [7]. This data-driven approach facilitates personalised therapeutic strategies, providing the continuous feedback loop necessary to automatically adjust treatments based on a patient’s unique physiological state.

In the context of respiratory health, Engin et al. [8] address the classification of lung sounds (LSs), identifying two principal limitations: the variability in respiratory cycle durations among individuals and the constraints of standard spectral representations. To address the issue of temporal variability, the study introduces an automatic respiratory cycle detection framework. Employing spectral energy density and dynamic time warping (DTW), the system segments lung sounds into individual breathing cycles, irrespective of their duration, thereby ensuring a consistent input for the classifier. This pre-processing step is of critical importance, as it eliminates the noise emanating from non-respiratory periods and standardises the data structure.

For classification, the authors have conducted a comparative analysis of various time–frequency representations, including spectrograms, scalograms, and Mel-spectrograms [9]. However, superior performance is achieved using Gammatonegrams, a representation based on the Gammatone filter bank, which mimics the human auditory system’s response. When fed into a DenseNet201 Convolutional Neural Network (CNN), these features achieve a classification accuracy of 97.3% across four classes (normal, rhonchi, fine crackle, and coarse crackle). Using a bio-inspired approach, the algorithm achieves greater robustness to noise and higher fidelity in the low-frequency ranges where pathological sounds often reside. The study also serves as a robust validation of transfer learning, demonstrating that CNN architectures pre-trained on natural images (like ImageNet) can be effectively repurposed for biomedical spectrograms.

## 4. The Cognitive Frontier: Agentic AI and the Trust Gap

The final pillar of this Special Issue addresses the most rapidly evolving and controversial technology in healthcare: generative AI. While Large Language Models (LLMs) offer unprecedented capabilities in summarising and generating medical text, they suffer from a critical flaw: hallucination.

Salehi et al. [10] provide a comprehensive review of LLM hallucinations in radiology, a domain where “plausible but incorrect” information can have lethal consequences. They cite hallucination rates of 8–15% in current medical imaging systems. The authors categorise these errors into a taxonomy: anatomical hallucinations (misidentifying structures), pathological hallucinations (inventing or missing lesions), and measurement hallucinations (generating false quantifications). The danger is compounded in Vision–Language Models (VLMs), where errors can originate in either the visual encoder or the language decoder, creating “dual-modal” failure points.

A key idea in Salehi et al. [10] is the support for Agentic AI. The authors argue that the current reliance on single-agent LLMs constitutes a fundamental architecture error for robust medical reasoning. Instead, they propose multi-agent systems where distinct LLM-based agents assume specialised roles within a structured workflow. In an Agentic AI framework, one agent might be responsible solely for information retrieval (searching the medical literature), another for summarisation, a third for analytical reasoning, and a fourth for quality control/judging. This “division of labour” mimics a clinical team, where a resident might draft a report, a senior radiologist reviews the images, and a multi-disciplinary team discusses the findings. By externalising the reasoning steps and creating validation checkpoints, Agentic AI can significantly reduce error propagation. The review cites evidence showing that Retrieval-Augmented Generation (RAG), a key component of Agentic systems, can reduce hallucination rates in specific tasks from 8% to 0%.

Furthermore, the authors discuss the integration of uncertainty quantification. In sophisticated multi-agent systems, agents do not just output text; they communicate mathematical confidence scores, allowing the “judge” agent to weigh inputs based on reliability. While acknowledging the computational costs and regulatory hurdles of such complex systems, the editorial perspective aligns with the authors’ conclusion: Agentic AI represents the necessary evolution from “chatbot” to “clinical partner”.

## 5. Methodological Synthesis: The Red Thread

When viewing these five articles in concert, several converging methodological trends emerge that define the current direction of biomedical signal and image processing.

For several years, the dominant narrative in AI has been “end-to-end learning”, where deep Neural Network learn features directly from raw data. However, the works in this Issue suggest a nuanced retreat from this dogma. Psathas et al. [6] demonstrate that evolutionary feature construction, creating explicit, mathematical features, can outperform raw-data deep learning in tasks with limited data, such as Parkinson’s monitoring. Similarly, Engin et al. [8] show that the choice of input representation (Gammatonegram vs. spectrogram) is a form of feature engineering that critically dictates model performance. Even in the deep learning-heavy work of Yamamoto and Kikuchi [4], the system relies on specific “lesion-aware” and “organ-aware” fine-tuning, effectively engineering the model’s attention towards clinically relevant features. From here, a clear lesson can be obtained: domain knowledge matters. Algorithms perform better when the data is pre-processed or represented in a way that reflects the underlying biology or physics.

A major bottleneck in biomedical AI is the high cost of expert annotation. Two papers in this issue offer powerful solutions to this problem. Yamamoto and Kikuchi’s [4] CLIP-based slice selector automates the linkage of text and images, potentially unlocking millions of archived CT scans for training. Engin et al.’s [8] automatic respiratory cycle detection removes the need for manual segmentation of lung sounds. These “AI for AI” tools, as algorithms designed to curate data for other algorithms, are essential for scaling biomedical AI beyond small, curated academic datasets to the messy reality of clinical archives.

Finally, the transition from Gómez et al.’s [3] radiomics review to Salehi et al.’s [10] Agentic AI review marks a shift in the purpose of biomedical AI. Radiomics is primarily a detection and classification tool (e.g., is this lymph node metastatic?). Agentic AI, however, is a reasoning tool. It attempts to synthesise information, check for consistency, and generate complex reports. As AI moves up the cognitive ladder, the metrics of success change. It is no longer sufficient to report an AUC or an F1-score; we must now evaluate hallucination rates, reasoning transparency, and agent collaboration dynamics.

## 6. Future Directions and Challenges

While the innovations presented in this Special Issue are impressive, they also show the challenges that remain before these technologies can be integrated as a standard of care.

Despite the abundance of healthcare data, high-quality, balanced datasets remain rare. Engin et al. [8] explicitly addressed class imbalance by curating a balanced dataset of 400 respiratory cycles, a luxury not afforded to most researchers. Gómez et al. [3] noted the variability in sample sizes and methodologies in rectal cancer studies. Future research must prioritise the development of federated learning networks and synthetic data generation (potentially using the Agentic workflows described by Salehi et al. [10]) to create robust, diverse datasets that reflect the global patient population.

There is an inherent tension between model performance and interpretability. The DenseNet201 used by Engin et al. [8] and the multi-agent systems described by Salehi et al. [10] are complex “black boxes”. Conversely, the Grammatical Evolution features generated by Psathas et al. [6] are mathematical expressions that can be inspected and understood. As regulatory bodies like the FDA and EMA tighten requirements for Software as a Medical Device (SaMD), the ability to explain why an algorithm made a decision will become as important as the accuracy of that decision. The explicit feature construction methods demonstrated here offer a promising path toward “White Box” AI or Explainable AI (XAI).

Salehi et al. [10] rightly point out that multi-agent systems challenge traditional regulatory frameworks. Who is liable when a “search agent” retrieves outdated guidelines and a “reasoning agent” acts upon them? The dynamic, non-deterministic nature of Agentic AI requires new validation protocols, such as continuous monitoring and “regulatory sandboxes”. Similarly, the practical deployment of the SmartGlove (Psathas et al. [6]) or the automatic lung sound classifier (Engin et al. [8]) requires integration into the Internet of Things (IoT) ecosystem, raising concerns about data privacy and real-time processing latency.

## 7. Conclusions

The articles in this Special Issue, “Machine Learning-Driven Innovations in Biomedical Signal and Image Processing”, paint a portrait of a field in rapid transformation. We are witnessing the convergence of distinct technological lineages: the rigorous signal processing of the 20th century is merging with the generative power of 21st-century foundation models.

From the SmartGlove that quantifies the chaotic tremors of Parkinson’s disease to the Gammatonegram that allows computers to “hear” pneumonia, we see a commitment to capturing the nuances of human physiology; from the CLIP-based automation of CT datasets to the Agentic orchestration of radiological reasoning, we see a commitment to scaling these insights to the level of population health.

The editorial board commends the authors for their invaluable contributions. Their work demonstrates that the “black box” of AI is slowly showing some transparency, not just by more data being available, but through better representations, smarter agents, and a higher recognition of the biological complexity they seek to model. As these technologies continue to mature, they promise to turn the noisy, high-dimensional data presented by biomedical signals into a clear, actionable context of clinical insight.
